# Clinic Atlanta Medical College

**Published:** 1874-01

**Authors:** 


### Clinic Atlanta Medical College.

### SURGICAL.

*W. F. Westmoreland, M.D., Professor of Surgery.*

E. A. COBLEIGH, Reporter.

Case 4.—Jane T., forty years of age, colored, washerwoman. This is the case of chronic eczema reported in full in the last number of Journal. December 17, 1873, she returned to clinic with arms entirely well, hands in an improved condition, beyond which the alkaline treatment would not force the disease, as was proved by persevering therein and finding no change effected. Ordered to procure a pair of kid gloves and fill with calamina preparata, then put them on the hands and wear them constantly with no other application. December 20th—Improving. Cleanse hands once daily, then apply fresh calamina with gloves as before. December 31st—Ordered to use diacylon ointment on hands and discontinue calamina. January 3d—Improving rapidly. Continue the ointment twice daily, and order no washing of the hands.

Case 8.—'Cajah G., colored, æt. twenty-six years, laborer. This case was also reported in last issue of Journal, being one of cold abscess pointing on outside of left thigh. Patient came before class, walking with crutches, on the 3d of January, having been under treatment since the opening of the abscess on November 8th, 1873. He is now almost free from pain, there being some little tenderness still remaining, but he can move the limb freely in all directions without difficulty. Great improvement in flesh and strength during the last few weeks. Abscess is still discharging a small quantity of sero-pus, but the probe demonstrates that this does not implicate the boney structures of the joint in the surrounding inflammation. If no relapse occurs the man will probably be well enough to resume his work in about two months, after a period of idleness dating back nine months or more.

Case 32.—December 17th. Lena A., negress, cook, æt. fifty-eight years. Has a large and rapidly extending phagadenic ulcer on left ankle, outer side, varicose veins coexisting with the sore, and probably causing it. Raw surface looks soft like bruised beef, fungous and congested granulations, and hemorrhage almost constant, being more profuse, and mingled with pus, when the ulcer is irritated by the clothing. Patient cannot walk, and complains of burning and acute lancinating pain in this region. Poultice of flax-seed meal was applied for three days, and then muriated tincture of iron was painted upon the sore once daily and roller bandage used after the application. January 3d—treatment continued up to date. Ulcer still as large as the palm of the hand, but diminished in size since first seen. Has been trying to walk, and the granulations are considerably congested, which was not the case three days ago. Persevere in the use of the tincture of iron and bandage, and keep perfectly quiet until cured.

Case 34.—December 17th, 1873. Edward W., white, æt. sixteen years, school-boy. This patient had congenital phymosis which was treated by amputation of the surplus amount of prepuce and closure of wound with silk suture. December 20th—came again before the class and reported parts healing rapidly, with no pain, little suppuration and very slight tumefaction.

Case 37.—Moses G., æt. thirty-five years, saddler, unmarried, negro. Came to the Clinic December 31st, 1873. Had syphilis a year ago, he says. Was taken with chills sixteen days ago, then followed fever and severe back-ache. Examination of penis shows that organ in a very filthy condition, but does not reveal the present existence of a chancre. A large bubo, however, was found in the left groin, and patient gave signs of commencing crural phlebitis, attributable to the inflammation in the groin and pressure upon the femoral vessels and nerves. The bubo was at once opened, the incision being carried entirely through the inflamed parts. No other treatment instituted.

Case 38.—December 31st. Romulus G., colored, twenty years of age, single, a blacksmith by trade, presented himself for treatment of gonorrhœal rheumatism. The case was interesting only from the peculiar conformation of the prepuce. No gonorrhœa was found to exist now, but the prepuce was half absent, he says from birth and is now adherent to the glans. The half which was found hung like an apron over the glans when the penis was not erected, and on stretching it with the thumb and fingers it spread out in the form of a bat with extended wings. No frænum whatever could be found and the urethra opened on under side of base of glans, a mere fissure where the canal ought to be, running forward to the usual place of opening, forming what is known as hyospadias. Rheumatism treated with the following:

℞.—Quiniæ Sulphatis . . . . . . gr. xxx.

Pulveris Opii . . . . . . . gr. iv.

Mix and make powders No. 8, one to be taken three times daily, and an ointment of lard and pepper to be made by patient and briskly rubbed over the painful parts of the limbs.

Case 39.—Viney J., æt. twenty-four years, negress, cook. Came to Clinic January 3d, 1873, stating that she had been sick about a year. She is lame in the left leg and shows signs of pain when walking, even with her crutch. In the left gluteal region was found six or eight fistulous openings, not very deep but from which sero-pus was discharging. These have been there nearly the entire duration of her illness. She is sensitive to touch on the hip. Says she has had very little pain in the knee, but refers all her suffering to the region of the hip joint. Probe failed to reach the bones of the articulation, or indeed any bony structure at all, and no definite diagnosis was made, though coxalgia is suspected. The parts were freely incised, and considerable pus escaped. No other treatment ordered; she will return to the next Clinic, when a more thorough examination will be made. This woman is a sister to 'Cajah. G., (Case No. 8), who was troubled somewhat as she is, leading us to suspect a vitiated condition of general system as being hereditary in the family.

---

### EYE AND EAR.

---

*A. W. Calhoun, M.D., Professor of Diseases of the Eye and Ear.*

---

Case 5.—Henry W., six years old, colored. This case was under Dr. Calhoun's treatment at Clinic, when last report was made. His disease was ophthalmia pustulosa, and on December 13th he had relapsed after apparent cure, because the remedies ordered for him were discontinued. He was put again upon the treatment first instituted, viz:

℞.—Hydrag. Oxidi Flav., . . . . . grs. iss.
Cerati Simplicis, . . . . . . ℨ. j.

Misce. Apply a small quantity to lower palpebral conjunctiva once daily, after cleansing the eye with warm water. Also dust calomel over the same surface once a day with a camel's hair brush, and drop in two or three drops of solution of sulphate of atropia, one grain to the ounce, *pro re nata.* Up to the last time he was seen—January 3d—the improvement was steady and rapid, and at that time he appeared to be entirely well. Treatment was, however, continued, to make sure of no recurrence of the trouble. Discharged.

CASE 12.—December 10th, 1873. Willie G., white, three and a half years of age. Brought before the class by his father, with blepharadinitis, with some sympathetic conjunctivitis. Edge of eye-lids thickly covered with concreted secretion of Meibomian glands, and showing a tendency to adhere to each other. Slight photophobia and lachrymation. Ordered the following treatment:

℞.—Hydrarg. Oxidi Rub. . . . . . grs. ij.
Cerati Simplicis . . . . . ʒ. j.

Misce. Thoroughly cleanse the eyes twice daily, removing with the finger-nail all scabs on the lids, then apply the ointment to the denuded surfaces. December 31st—improving. Excoriations on cheeks, conjunctivitis and blepharadinitis all markedly better. Continue treatment.

CASE 13.—December 31st. N. B. B., negro, hackman, thirty-six years old, comes from Minnesota. This man has chronic conjunctivitis of eight years standing. Has exacerbations, being sometimes nearly well and then relapsing. Patient was put in charge of one of the students, who would apply, once a day, with a camel's hair pencil, to the inflamed parts, a solution of nitrate of silver—grs. x to ℥j of water.

CASE 14.—January 3d. Aldis K., white æt. twenty-seven years. This man has blepharadinitis, with other symptoms, as in Case 12, but slightly marked. Disease is of several years duration. Ordered,

℞.—Hydrarg. Oxidi Rub. . . . . . grs. ij.
Unguenti Aquæ Rosæ, . . . . . ʒ. j.

Misce. Use as directed for Case 12.

CASE 15.—Percy Christian, white, æt. fourteen years. Blepharadinitis. Had conjunctivitis scrofulosa for years, upon which affection the blepharadinitis depends, in this instance. Ordered:

℞.—Hydrarg. Oxidi Flav. . . . . . grs. iij.
Unguenti Aquæ Rosæ, . . . . ℨ. ij.

Misce. Use as ordered for Case 12, and continue the application to the eyes for some weeks after apparent cure. Also take plenty of exercise in the open air and partake of rich, nourishing food to improve the general condition.

Case 16.—January 3d, 1874. Benjamin G., twenty-two years old, colored, school-teacher. Has opacity of cornea in both eyes, the result of keratitis and ulceration several years ago. Perforation took place from an ulcer of right cornea, and gave rise to an anterior synechia in that eye. In left eye the opacity is large and situated directly in front of the pupil, obstructing the sight in that eye. The right is still useful, as the opacity is out of the line of vision and the pupil not very much distorted by the synechia. Iridectomy was performed on the left for the purpose of making an artificial pupil to one side of the opaque spot.

---

### MEDICAL.

*J. G. Westmoreland, M.D., Prof. Materia Medica and Therapeutics.*

J. G. McKENEY, Reporter.

Case 1.—November 21, 1873. D. McB., a white male, æt. twenty-seven years. Has a soft, fluctuating tumor near elbow of right arm, one under right inferior maxilla, somewhat indurated, and slight redness and tumefaction of the left ankle. The history given by the patient shows that over a year ago he contracted syphilis. The chancres soon healed and the bubo disappeared without suppuration. In a few months sore throat came on, occasionally disappearing, but only gave way finally on the appearance of the subcutaneous disturbance now present.

We have in this man, young gentlemen, a representative case of syphilitic contamination. Its history furnishes us with the usual course of the disease. Chancres which give very little trouble or uneasiness to the subject are followed by most serious symptoms. The Hunterian or hard chancre is of this description, and is known by its indurated base, sometimes giving the sensation, when taken between the fingers, of a shot beneath the surface, and its tendency to heal readily without destruction of the tissues in which it is located—the induration often remaining after the ulceration of the surface disappears, particularly if catalytics in sufficient amount to eliminate the virus are not given. In this case, as in many others, the chancre healed readily, and the patient supposed himself well of the pock. For two months no symptom of the disease was felt, and when in the form of ulcerated tonsils the secondary symptoms appeared, he was not prepared to recognize them as the result of the disease he had contracted three months previously. We must remember that too often this false security, and ignorance of the fact that the throat and skin most frequently exhibit secondary symptoms, lead to just such results as we have presented to-day in this case. Treated, perhaps, as ordinary sore throat "from cold," it disappeared only by giving place to other and more serious results.

Without elimination of the syphilitic virus, still more dangerous effects may be expected. The periosteum or internal vital organs may soon give evidences of disease, and terminate the existence of the unfortunate man.

Do not think, young gentlemen, that this is an over-drawn picture; for doubtless many perish from supposed natural organic disease originating in the deposit of syphilitic virus.

The practical question now presents itself: How shall these results be prevented? How shall syphilis be cured? First, let me impress you with the fact that hard chancre or true syphilis, unlike soft chancre or chancroid, is inconsiderable in its first developments. No phagadenic ulcer leading to destruction of tissue is found. An ulcer so small as scarcely to be noticed, and, in females, sometimes not discovered by the patient, is often all of primary symptoms that appear in cases which, without proper treatment, result in physical ruin to the subjects and their offspring.

Had the patient before us taken liberally of mercury and and iodine for a month or two after the chancres appeared, the virus would have been elimenated, and none of the results which followed would have been experienced. Now a different course is required. Mercury is not perhaps necessary at this stage of the disease. Iodine, which, up to the third month proves effective in comparatively small quantity, say ten grains of the iodide of potassium must now be given in the dose of more than three times this amount. We will, however, commence the treatment by giving twenty-two or twenty-three grains three times a day. The dose may be increased to forty and even sixty grains, as the stomach is found to tolerate it. The quantity taken should be dissolved in half a pint of water or flax-seed tea. For convenience I make the following:

℞.—Iodide Potassium, . . . . . ℥ jss.

Syrup Sarsaparilla, . . . . . Oj.

Dissolve, and direct one tablespoonful three times a day in half a pint flax-seed tea.

As sarsaparilla is also a catalytic of syphilitic virus, its addition to the iodide is desirable, and in the form of syrup serves also as an excipient.

Case 2.—Emma Green, colored woman, æt. twenty-eight, was attacked, not violently, two months since with symptoms of ordinary "bad cold;" has had occasional cough since, with slight expectoration of mucus. The feet are slightly œdemetous, and some tenderness on pressure is found in the right side of the chest. On percussing you will perceive a difference between the right and left sides. While the left is resonant, the right gives a dull sound, as if more solid structure is contained within the walls. The sounds of respiration in auscultation give also a marked difference in the two sides of the chest. The ordinary vesicular murmur is heard in the left lung, while it is scarcely perceptible in the right, particularly at the apex. These physical signs lead to the conclusion that the right lung is more solid than is natural, and that the usual amount of air does not enter it in respiration. This condition may be the result of different causes. The general symptoms, history of the case, etc., aid in determining this question. Sudden congestion and acute inflammation of the lung would lead to solidity of the organ, and give the physical signs we discover in this case. The general symptoms and history, however, will not warrant such conclusion. The fever and dyspnœa attendant upon sudden interference with the ingress of air into the lung from congestion and inflammation are not present. General debility and emaciation are the prominent general symptoms, and the history given shows slow progress of development, leading to the suspicion of tuberculous origin. Again, the apex being that part of the lung more subject to the deposit of tubercle, the opinion is strengthened by the more decided dullness on percussion, and less vesicular respiration just below the clavicle.

For the present, I shall content myself with an attempt to give general vigor to the body, by making the blood more nutritious, and the nervous power more active. A strumous constitution, one which is prone to the development of tuberculous or scrofulous matter, requires invigorating means above all others. I, therefore, direct this woman to take regularly nourishing and calorifacient articles of food, such as milk, butter, meat, etc., whether the appetite requires it or not. If the digestive organs fail to prepare it, remedies directed to their direct invigoration must be given. Some opiate preparation must be taken once daily for a few days, or until her return.

---

### GYNÆCOLOGICAL.

*V. H. Taliaferro, M.D., Professor of Gynæcology.*

---

H. F. SCOTT, Reporter.

---

Case 1.—Fanny S., æt. twenty-six, colored, house-servant, married, two children, four years, and six weeks, respectively. No abortion; indisposed since last labor; in walking, pain in back, loins and hypogaster; some irritability of bladder, with frequent micturition. Menses established at fifteen; regular until last pregnancy, since which none. *Vaginal touch,* a well-defined tumor accessible through posterior cul-de-sac. *Rectal touch,* nodulated tumor and great tenderness in recto-uterine space. *Speculum* caused pain; cervix greatly enlarged, with congestion, granular ulceration of os; profuse muco-purulent discharge. *Sound* gave pain, passed in normal direction; uterine cavity $3\frac{3}{4}$ inches. Diagnosis: Subserous fibroid upon posterior wall of uterus; chronic corporeal-endometritis. *Treatment:* Tinct. Iodine applied to entire vaginal cervix; Fl. Ext. Ergot, 30 drops three times daily.

November 8th. Still great tenderness on rectal touch; cervix slightly improved. *Treatment:* Cloth tent coated with an ointment of Iodine, (strength of Lugol's Caustic Iodine) passed into uterine cavity; cotton and glycerine pessary applied. Continue ergot with Potass. Iod. gr. v, three times daily.

Case 2.—November 22d. Mrs. Carrie B., æt. forty-three, white, laundress, widow, five children, aged fifteen to three, respectively; no abortions. Medium size, dark complexion, muscular, sleeps well; no pain, mind cheerful; complains of globus hystericus, indigestion, occasional oppressed respiration; menses regular, last three days, with increased globus. *Taxis,* vaginal wall flaccid; cervix enlarged, hard, granular, looking forwards, rather low; fundus resting against the rectum; entire uterus enlarged and sensitive. *Speculum:* Cervix much congested, enlarged villi and loss of epithelium at the os. *Sound* elicits tenderness of os, passes towards rectum, no obstruction at internal os, slight flow of blood; cavity of uterus three inches. *Diagnosis:* sub-involution and retroversion. *Treatment:* Cloth tent saturated with Churchill's Tinct. Iodine and coated with ointment of equa strength; cotton and glycerine pessary. November 29th. Considerably better; there is now right-latero-version; less congestion and ulceration. December 13th. Still right-latero-version; very little leucorrhœa; ulceration gone.

CASE 3.—November 8th. Julia A., æt. forty-five, colored, cook. Menstruated at fourteen, five children, aged twenty-five to nine years, respectively. No abortion; complains for three months of pain in hypogastrium, hips and back; vertigo; imagines her womb comes out of the vulva; nutrition good, though she says she has lost flesh. *Examination:* Growth three inches long, from anterior lip of the uterus protrudes between the labiæ. *Diagnosis:* Hypertrophic elongation anterior lip of uterus. November 15th. Patient in left lateral position, Dr. J. C. Connally assisting, the hypertrophic elongation was removed; case progressed favorably until the sixth day, when violent secondary hemorrhage occurred. December 6th. Wound cicatrized; there remains endometritis, which is receiving treatment.

CASE 4.–Martha S., æt. eighteen, colored, laundress; menstruated about fifteen; fourteen months married; no conception; complains of pain in right ovaric region and back, more or less constantly, especially distressing during menstruation, which is regular every twenty-eight days, continuing four to fourteen days. *Examination:* Virginal cervix in normal position; fundus slightly anteflexed; entire organ highly sensitive; profuse viscid leucorrhea, rust colored. *Bimanual taxis;* great tenderness of entire uterus. *Sound* gives much pain; no obstruction at inner os; direction slightly forward; flow of blood on withdrawal; cavity two and three-fourths inches. *Diagnosis:* General endometritis. *Treatment:* Cantharidal-collodion introduced upon cloth tent, and freely painted over the vaginal cervix and *cul-de-sacs.* *Remark:* The blister within the uterus and upon the cervix is for purpose of depletion, and relief of congestion. For this purpose it will prove decidedly effective, after which the usual treatment for endometritis may be instituted with safety.

Case 5—December 6th. Mary C., æt. thirty-five, colored, laundress, large, robust, well developed. Menstruated at fourteen; married fifteen years; ten children; fourteen to three years respectively. Abortion at six months, four months ago, when she was confined two months to bed; health bad for three years; complains of hypogaster and back; also vertigo, and smothering on exertion. Heart beats violently; acute rheumatism eight or nine years ago. Menses more protracted and profuse than formerly, with great pain. No leucorrhœa. *Examination:* Cervix directed slightly forward; a considerable rounded mass against posterior *cul-de-sac;* body uterus fixed and somewhat prolapsed. *Rectal touch:* Rounded mass clearly defined in Douglas' pouch. *Speculum:* Congestion, cervix and os. *Sound* passes with difficulty and pain towards hollow of sacrum; cavity four inches. *Knee-elbow position:* Can press the rounded mass towards pubis; sound in uterus follows with it. *Diagnosis:* Hypertrophy of posterior wall of uterus, consequent retroflexion, and sub-involution. *Treatment:* Cervix and cavity thoroughly cleansed; cloth tent with Churchill's caustic iodine and coated with ointment to cavity; cervix painted with same and dressed with cotton and glycerine. Constitutionally:

℞.—Potassii Iodid, . . . . . . . gr. v

Extract Ergot, Fluid, . . . . . ʒss.

Aquæ, . . . . . . . . . ℥j.

M. Ft. Haust. Sig. To be repeated half-hour before each meal.

December 20th. Still excessive action of the heart, but more moderate; marked improvement in uterus; depth of cavity three and three-fourth inches; uterus almost in normal position; sound turns now upwards instead of downwards; the rounded mass has disappeared from Douglas' fossa. *Treatment:* Cloth tent saturated with carbolic acid and coated with the ointment of iodine. Cotton and glycerine dressing, and other prescription continued.

December 27th. Improved; local and general treatment continued.

